# Economic evaluation of a dedicated cardiac resynchronisation therapy preassessment clinic

**DOI:** 10.1136/openhrt-2020-001249

**Published:** 2020-07-20

**Authors:** Baldeep Singh Sidhu, Tiago Rua, Justin Gould, Bradley Porter, Benjamin Sieniewicz, Steven Niederer, Christopher Aldo Rinaldi, Gerald Carr-White

**Affiliations:** 1 School of Biomedical Engineering and Imaging Sciences, King's College London, London, United Kingdom; 2 Cardiology Department, Guy's and St Thomas NHS Foundation Trust, London, United Kingdom

**Keywords:** pacemakers, cardiac resynchronisation therapy, delivery of care

## Abstract

**Introduction:**

Patient evaluation before cardiac resynchronisation therapy (CRT) remains heterogeneous across centres and it is suspected a proportion of patients with unfavourable characteristics proceed to implantation. We developed a unique CRT preassessment clinic (CRT PAC) to act as a final review for patients already considered for CRT. We hypothesised that this clinic would identify some patients unsuitable for CRT through updated investigations and review. The purpose of this analysis was to determine whether the CRT PAC led to savings for the National Health Service (NHS).

**Methods:**

A decision tree model was made to evaluate two clinical pathways; (1) standard of care where all patients initially seen in an outpatient cardiology clinic proceeded directly to CRT and (2) management of patients in CRT PAC.

**Results:**

244 patients were reviewed in the CRT PAC; 184 patients were eligible to proceed directly for implantation and 48 patients did not meet consensus guidelines for CRT so were not implanted. Following CRT, 82.4% of patients had improvement in their clinical composite score and 57.7% had reduction in left ventricular end-systolic volume ≥15%. Using the decision tree model, by reviewing patients in the CRT PAC, the total savings for the NHS was £966 880. Taking into consideration the additional cost of the clinic and by applying this model structure throughout the NHS, the potential savings could be as much as £39 million.

**Conclusions:**

CRT PAC appropriately selects patients and leads to substantial savings for the NHS. Adopting this clinic across the NHS has the potential to save £39 million.

**Key questions**

**What is already known about this subject?**

- Cardiac resynchronisation therapy (CRT) is important in the management of patients with symptomatic heart failure, electrical dyssynchrony and severe left ventricular systolic impairment. It reduces morbidity and mortality but nearly 30%–40% of patients will fail to respond. There are many reasons why patients are CRT non-responders and it is believed that a proportion of patients are implanted with unfavourable characteristics such as narrow QRS duration, whereby CRT is inappropriate. The exact number of these patients is unknown as there is insufficient data in the published literature. Additionally, CRT has considerable cost implications for the healthcare system in terms of procedural costs, follow-up requirements and the possible need for future device revisions. Given these considerations, only patients who completely satisfy guidelines should proceed for CRT. However, currently there is no preassessment process to ensure that guidelines are strictly followed.

**Key questions**

**What does this study add?**

- This study provides an economic evaluation for patients attending a dedicated and specialised CRT preassessment clinic (CRT PAC). All patients reviewed by a consultant cardiologist in an outpatient cardiology clinic and who thought CRT was appropriate were subsequently seen in the CRT PAC prior to intervention. This clinic was developed to standardise the process for CRT implantations, to ensure only patients who fulfilled consensus guidelines proceeded to implantation. To our knowledge, this is the first ever clinic developed to thoroughly evaluate patients pre-CRT. We have shown that 82.4% of patients had an improvement in their clinical composite score and 57.7% had a reduction in their left ventricular end-systolic volume of ≥15%. We developed a decision tree model to compare the costs of running this specialised clinic with one that did not have a preassessment clinic but instead patients originally referred for CRT would proceed directly to implantation. Overall, the model demonstrated that the CRT PAC led to savings for the National Health Service (NHS) of £1 056 302, representing a saving of 20%. Furthermore, if this model was adopted in more centres across the NHS, the potential savings could be £39 million.

**How might this impact on clinical practice?**

- This analysis has the potential to considerably change the way we care for patients with heart failure and implant CRT in the UK. Currently, the incidence of heart failure and CRT are both increasing, at a considerable cost to the NHS. We have shown that a CRT PAC can appropriately select patients for implantation and result in favourable left ventricular remodelling at follow-up. Furthermore, this clinic led to substantial savings for our Trust and has the potential to lead to considerable savings across the NHS.

## Introduction

Cardiac resynchronisation therapy (CRT) is important in the management of patients with symptomatic heart failure (HF), who have evidence of electrical dyssynchrony and severe left ventricular (LV) systolic impairment.^1^ It reduces morbidity and mortality and consequently implantations have continued to rise, with new and replacement CRT devices estimated at 541 per million population in the UK between 2015 and 2016.^1–3^ Despite these benefits, nearly 30% of patients fail to respond with CRT^4 5^ and coupled with the requirement for regular pacing checks, generator changes and rising device-related complications, such devices should only be implanted if patients fully satisfy consensus guidelines.^1 2 6^ Indeed, careful patient selection is important to maximise CRT response and patients inappropriately referred with unfavourable characteristics such as a narrow QRS duration may account for a proportion of those labelled as ‘CRT non-responders’.^7^ Furthermore, the evaluation of patients before CRT is heterogeneous across centres; some centres carry out additional investigations to those recommended in consensus guidelines, some centres discuss all cases in a multidisciplinary meeting before proceeding to implantation and there is a varying follow-up period after establishing the patient on optimal medical therapy before deciding on CRT. Given the heterogeneity in the preprocedural patient evaluation, we developed a standardised approach for all CRT implantations by reviewing patients in a unique and dedicated CRT preassessment clinic (CRT PAC). This clinic acted as a final review for patients to ensure only those who fully satisfied consensus guidelines proceeded to implantation. We hypothesised that since this clinic would scrutinise referrals and patients would have updated investigations, a proportion of patients would be unsuitable for CRT and thus this would result in cost savings. Therefore, the purpose of this analysis was to determine whether the CRT PAC led to savings for the National Health Service (NHS).

## Methods

The CRT PAC was established to standardise the referral process for CRT and ensure only suitable patients proceeded to implantation. All patients who had been reviewed by a consultant cardiologist in an outpatient clinic and who thought CRT was appropriate based on their symptoms and previous investigations were seen again in the CRT PAC prior to undergoing device therapy. This clinic took place at a busy tertiary centre; Guy’s and St Thomas’ NHS Foundation Trust (GSTT). During this clinic, patients underwent up-to-date investigations including an ECG, echocardiogram and cardiac magnetic resonance scan. They were reviewed by a consultant cardiologist specialising in HF and only patients who satisfied consensus guidelines for CRT^1 2 8^ were implanted. The decision to implant a CRT-pacemaker (CRT-P) or defibrillator (CRT-D) was according to international guidelines. Patients with advanced HF, New York Heart Association (NYHA) IV or poor prognosis were implanted with a CRT-P. Patients with a presumed pacing induced cardiomyopathy were more likely to receive an upgrade to a CRT-P. Additionally, a de novo CRT was considered for patients with HF with a reduced ejection fraction and an expected high percentage of ventricular pacing. Patients requiring further medical optimisation prior to device therapy were re-evaluated in the CRT PAC to determine whether they should have CRT. Difficult cases with borderline characteristics for CRT implantation were discussed at a multidisciplinary team meeting. All patients were followed up to determine their progress, including those who were rejected.

A prospective database of consecutive patients attending the CRT PAC between 2014 and 2018 was analysed. We used a combination of clinical improvement and evidence of reverse LV remodelling to define CRT response. Patients were considered CRT responders if at 6 months (1) they had improvement in their clinical composite score (CCS) consisting of survival to follow-up, no hospitalisations with decompensated HF, improvement of ≥1 NYHA Functional class or improvement in a patient’s global assessment^9^ or (2) reduction in left ventricular end-systolic volume (LVESV) of ≥15% at follow-up. The study received institutional approval from GSTT.

### Cost analysis

A decision tree model was constructed (Microsoft Excel 2013) to estimate the cost associated with the CRT PAC. The model considered the evaluation of two clinical pathways in the management of patients requiring CRT implantation: (1) standard of care with no specialised clinic where patients initially seen in an outpatient consultant cardiology clinic and referred for CRT would proceed directly to implantation; and (2) management of patients attending a specialised CRT PAC. The standard of care pathway is currently used in most centres across the UK. All costs considered in the model were based on the NHS and personal social services’ perspective (table 1). The model’s time horizon was consistent with the follow-up period of this study. The specialised clinic accounted for the cost for each investigation and review by a consultant cardiologist, which was estimated at £561 per appointment. The CRT implantation cost was calculated as the sum of the cost for each device, cost for the cardiac catheter laboratory staff during the procedure and cost for an average hospital inpatient admission following CRT. This amount was estimated by averaging the cost of five patients who underwent each intervention at GSTT to give the following values; CRT-D £27 413 and CRT-P £10 542. The model did not account for the cost of device clinic follow-up appointments but did consider follow-up costs associated with HF admission events. One-way deterministic sensitivity analysis was performed around the key model parameter, the unit cost of running the specialised CRT PAC.

**Table 1:** Unit cost estimates

	Type of National Health Service event	Estimated mean cost (in £)
Clinic	No specialised clinic	0
	Cardiac resynchronisation therapy preassessment clinic	561
Cardiac resynchronisation therapy procedure	Cardiac resynchronisation therapy defibrillator	27 413
	Cardiac resynchronisation therapy pacemaker	10 542
	No CRT implantation	0
Follow-up costs	Hospital admissions due to heart failure events	7130

The mean unit cost for the cardiac resynchronisation therapy (CRT) preassessment clinic (£561) was estimated based on the utilisation of: (i) new outpatient appointment for cardiology (244/244 participants with an estimated unit cost of £200 per new outpatient consultant appointment); (ii) cardiac magnetic resonance (CMR) imaging (125/244 participants, unit cost of £544 based on unbundled CMR tariff) and (iii) echocardiogram (244/244 participants, unit cost of £82 for unbundled echocardiography). The mean unit costs for the cardiac resynchronisation therapy implantation procedures were the sum of the cost of the device, cost for the cardiac catheter laboratory staff and average hospital admission length. This amount was estimated by averaging the cost of five patients who underwent each intervention at our institution. The mean unit cost of hospital admissions during the follow-up period of 1 year was based on the costs incurred in the ‘specialised clinic’ group.

### Statistical analysis

The results are presented as mean±SD for normally distributed variables and as median and IQR for non-normally distributed variables. The independent-samples t-test was used to compare normally distributed continuous variables; otherwise the Mann-Whitney U test was used. A p-value of <0.05 was considered statistically significant for all tests. All statistical analyses were performed using Statistical Package for the Social Sciences Statistics Version 24.0.0.1.

## Results

### Patient demographics

A total of 244 patients were seen in the CRT PAC between 2014 and 2018. Patient demographics are provided in table 2. Patients were predominately male (72.1%) with a mean age of 70.6±10.8 years, mean NYHA functional class of 2.5±0.7 and left ventricular ejection faction (LVEF) 31.9%±10.2%. The majority of patients had left bundle branch block (56.1%) with a mean QRS duration of 156.7±28.2 ms.

**Table 2:** Baseline patient demographics

Variable	Overall (n=244)	Eligible for CRT (n=184)	Ineligible for CRT (n=60)	P value
Characteristics
Age, ±SD	70.6±10.8	70.8±10.6	69.8±11.5	0.576
Female, N (%)	68 (27.9)	44 (23.9)	24 (40.0)	0.016
Ischaemic aetiology, N (%)	124 (50.8)	97 (52.7)	27 (45.0)	0.299
Comorbidities, N (%)
Hypertension	87 (35.7)	69 (37.50)	18 (30.0)	0.292
Diabetes mellitus	70 (28.7)	58 (31.5)	12 (20.0)	0.087
Chronic kidney disease	60 (23.8)	43 (23.4)	17 (28.3)	0.438
New York Heart Association Functional class, ±SD	2.5±0.7	2.6±0.7	2.4±0.7	0.033
QRS duration, ±SD	156.7±28.2	161.5±26.7	142.0±27.8	<0.001
Left bundle branch block, N (%)	137 (56.1)	109 (59.2)	28 (46.7)	0.088
Echocardiography, ±SD
Left ventricular ejection fraction	31.9±10.2	29.1±8.2	40.5±11.2	<0.001
Left ventricular end-diastolic volume	188.8±78.7	203.7±80.5	140.8±48.0	<0.001
Left ventricular end-systolic volume	129.7±55.8	143.3±53.7	85.8±53.7	<0.001

CRT, cardiac resynchronisation therapy; N, number.

### Outcomes of patients attending CRT PAC

Following CRT PAC review, 184 (76.0%) patients met consensus guidelines for CRT and were recommended to proceed directly for implantation. However, in 16 patients, CRT was not possible due to; death before the procedure (n=2), unable to place the LV lead (n=5) or the patient declined intervention (n=9). The remaining 168 patients proceeded to implantation; 114 CRT-D and 54 CRT-P. After a median follow-up of 6 months, 82.4% of patients had an improvement in their CCS and 57.7% had a reduction in LVESV ≥15%. In patients who had a reduction in LVESV ≥15%, only 7.0% of patients failed to show an improvement in their CCS. Additionally, in patients who had an improvement in their CCS, 38.9% of patients did not display a reduction in LVESV ≥15%.

Following CRT PAC review, 60 (23.8%) patients did not meet consensus guidelines for CRT and this was often due to a combination of reasons; LVEF >35% (n=42), need to further optimise medical therapy (n=17), QRS duration <120 ms (n=9), NYHA functional class I (n=7) and end-stage HF (n=2). In the 42 patients with a LVEF >35%, there was a significant improvement in their LVEF at CRT PAC compared with 6 months previously, which had initially prompted CRT referral (45.1 vs 34.1%; p<0.001) and this was likely to have occurred due to improved medical management prior to CRT PAC review. In patients with a QRS duration of <120 ms, four patients had a QRS duration of ≥120 ms when initially referred for CRT and the finding of a narrow QRS during CRT PAC prompted a prolonged period of monitoring revealing a predominantly narrow QRS duration which made them ineligible for CRT. The other five patients were considered for CRT upgrade and they had a narrow intrinsic QRS duration, broad paced QRS duration and high ventricular pacing burden. In these patients, their devices were reprogrammed to reduce right ventricular pacing and their beta-blockers reduced, leading to a right ventricular pacing burden of <40% at follow-up making them ineligible.

Overall, of the 60 patients initially found to be ineligible for CRT, 12 patients proceeded to implantation during the 11-month (IQR 5–27 months) follow-up period. Patients underwent device implantation for a variety of reasons; persisting severe LV systolic impairment despite being optimised on medical therapy and deterioration in symptoms when previously asymptomatic. In the remaining 48 patients who did not undergo implantation, two patients who were initially rejected for CRT due to end-stage HF were admitted to hospital and died. There were no other hospital admissions for decompensated HF nor deaths in the other patients turned down for CRT.

### Cost analysis

**Figure 1:**
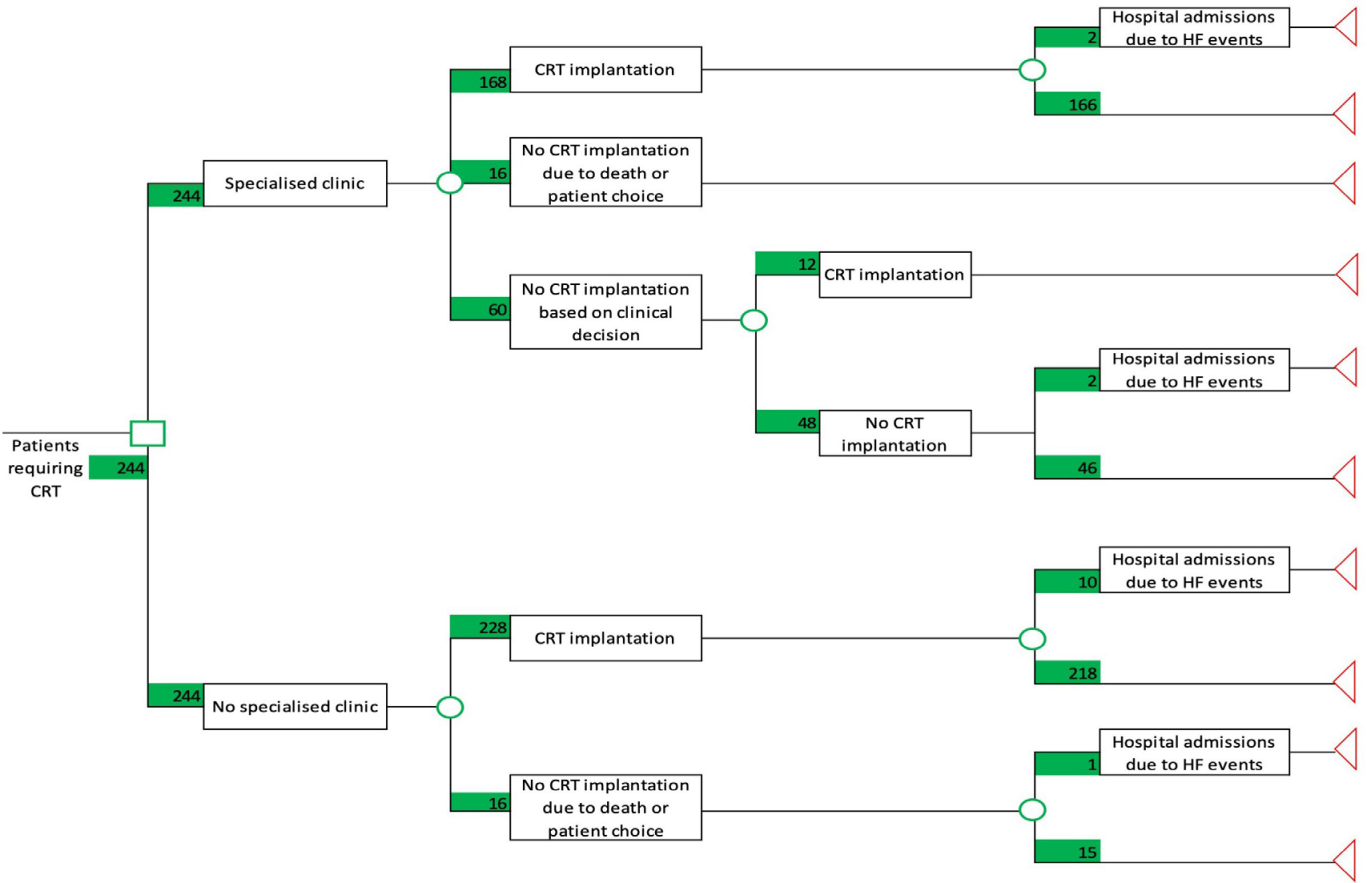
Decision tree associated with the management of patients requiring cardiac resynchronisation therapy with and without a preassessment clinic This decision tree model was used to estimate the cost of the cardiac resynchronisation therapy preassessment clinic (CRT PAC). The specialised clinic refers to the CRT PAC. Patients requiring CRT with no specialised clinic were considered to either proceed to CRT implantation or not. Similarly, patients attending the specialised clinic had the same options but as a result of the CRT PAC review, 60 patients were unsuitable to proceed directly to CRT. During the follow-up period, 12 patients underwent device implantation but 48 patients did not. CRT, cardiac resynchronisation therapy, HF, heart failure.

A decision tree model was constructed (figure 1) from this prospective study of 244 participants. Patients requiring CRT with no specialised CRT PAC were considered to either proceed to CRT implantation (n=228) or not proceed to CRT implantation (n=16) because of failure to implant the LV lead, death before the procedure or the patient refused the intervention. Similarly, participants attending the specialised CRT PAC clinic were considered to have the same options. However, based on reviews during the specialised CRT PAC clinic, a higher proportion of patients were considered not to proceed to CRT (n=60). Based on the study’s 11 month (IQR 5–27 months) follow-up period, a proportion of these patients underwent device implantation (n=12), while the remaining did not undergo any procedure (n=48).

Taking account of the model structure and unit costs considered, the total cost for the NHS and the mean cost per patient for both strategies were calculated and are presented in table 3. The implementation of a specialised CRT PAC is associated with an estimated cost-saving of £1 056 302 for the entire 244 patient cohort, or else, a saving of £4329 per patient which represents a savings of 20% compared with standard of care. Furthermore, these savings are likely to be underestimated as the required number of pacemaker clinic follow-up appointments is lower in the CRT PAC arm. Assuming a lower (£350) and higher (£1000) unit cost, the intervention led to cost-savings per patient of £4540 (21%) and £3890 (18%), respectively. Hence, even when a considerable increase (78%) of the cost of running the CRT PAC was considered, this intervention was still associated with significant cost-savings from the NHS and Personal Social Services’ perspective.

**Table 3:** Total costs and mean cost per patient associated with both clinical strategies

Strategy	Total cost for the National Health Service	Total mean cost per patient
No CRT PAC	£5 288 257	£21 673
CRT PAC	£4 231 955	£17 344
Potential cost-savings	£1 056 302	£4329

CRT PAC, cardiac resynchronisation therapy preassessment clinic.

## Discussion

We present our real-world data of a dedicated and unique CRT PAC designed to ensure that only appropriate patients were implanted with CRT. The main findings from this analysis include:

1. Following CRT, 82.4% of patients had an improvement in their CCS and 57.7% of patients had an improvement in LVESV of ≥15%.
2. There was a cost saving of £1 056 302 for the entire cohort of 244 patients, which represented a 20% saving compared with no specialist clinic.

### Improving patient care

A major strength of this clinic was the ability to perform up-to-date investigations and regular patient reviews which when combined led to a reduction in CRT implantations and savings for the NHS. CRT optimisation clinics have been previously described and helped to improve patient symptoms following CRT.^10^ However, to our knowledge, no clinics have been developed before implantation to ensure that only those patients who fully satisfy consensus guidelines proceed to CRT. Furthermore, the number of patients who receive CRT inappropriately is unknown and based on our analysis is likely to be significant. These inappropriate referrals may represent a proportion of patients labelled as ‘CRT non-responders’ which was hypothesised in previous work.^11^ Therefore, although the CRT PAC did not use new or additional criteria to select suitable patients for CRT, the updated investigations and strict evidence-based approach led to savings and satisfactory patient outcomes.

### Importance of preassessment clinics

The purpose of developing a preassessment clinic is to select appropriate patients, reduce hospital admission lengths through patient optimisation, suitably schedule theatre times and therefore lead to cost savings.^12^ This clinic structure is predominantly used for surgical procedures with preassessment clinics an integral step in improving service productivity and resulting in cost savings prior to any cardiac surgery.^13^ Despite the range of invasive cardiac procedures, there is a relative lack of cardiology preassessment clinics. A notable exception to this is the development of valve clinics used to review both patients with native valve disease and those following valve intervention.^14^ Ionescu *et al* showed that a specialised valve clinic improved patient care and led to savings in healthcare costs.^14^ Adopting similar preassessment clinics prior to CRT is required given the rising incidence of HF and CRT implantations.^1^ Patients with symptomatic HF being considered for CRT are an older, complex patient group with multiple comorbidities who would likely benefit from a preassessment clinic.^4^ In our experience, having a CRT PAC to thoroughly assess these patients is extremely useful and facilitates a detailed assessment, discussions regarding the indications for device therapy and type of device, which is particularly important given emerging therapies.^15^ Our analysis also showed that in those eligible for CRT, nine (5.4%) patients declined intervention and this therefore avoided unnecessary cancellations enabling a better use of resources.

### Potential savings for the NHS

Delivering excellent care for patients with HF in the NHS has become increasingly difficult due to financial constraints, an ageing population and the increasing incidence of HF. The All Party Parliamentary Group on Heart Disease states that HF accounts for 2% of the NHS budget, estimated at £2 billion a year.^16^ Given the management of HF is diverse, a range of strategies should be developed to manage these patients and potentially result in savings for an already burdened healthcare system. Specialist nurse-led HF clinics have proven to be effective at improving patient care and reducing costs.^17^ We have demonstrated that the CRT PAC led to substantial savings for the NHS, estimated at £1 056 302 for the 244 patient cohort. While a potential alternative to this clinic would be to discuss every CRT case in a multidisciplinary meeting, we feel that the importance of up-to-date investigations was integral in deeming patients unsuitable for CRT and a clinic would facilitate detailed discussions with patients which may then reduce unnecessary procedural cancellations.

The CRT PAC resulted in additional patient and cost savings which were not accounted for by this analysis. Overall, 48 patients did not receive CRT and this resulted in procedural savings as previously discussed and since these patients did not need regular pacemaker clinic reviews, usually very 6–12 months, additional savings were made. Furthermore, in these patients, there would be no need for battery changes with the associated procedural and hospitalisation costs. Additionally, no device revisions would be required, particularly important given the incidence of device-related infections and lead complications.^6^ Therefore, although we have shown the CRT PAC results in considerable procedural savings, there are further ‘downstream’ effects which positively affect patients and results in further healthcare savings.

According to the NHS Reference costs from 2017 to 2018, a total of 9105 implantations of cardioverter-defibrillator with CRT (codes EY01A, EY01B) and biventricular pacemakers (codes EY03Z, EY04A, EY04B) were performed in the NHS in 1 year.^18^ If the CRT PAC structure were introduced in more centres and carried out prior to these procedures, it is estimated that there would be a total cost savings for the NHS of over £39 million, while optimising patient care.

### Study limitations

This is a single-centre, observational study and the results may not necessarily represent the overall burden to the NHS. The total number of patients inappropriately implanted with CRT is unknown and is likely to vary from centre to centre thus affecting the economic cost evaluation. Additionally, some centres review every CRT implantation in a multidisciplinary team meeting which may further reduce inappropriate referrals and thus reduce the overall saving resulting from a CRT PAC. It is conceivable that savings could potentially be reduced in smaller volume centres with fewer operators and the CRT PAC is more useful in high-volume centres with multiple operators. The costings for device implantation were not collected prospectively which may have led to differences in the overall estimated cost of CRT implantation.

## Conclusion

We have shown that a CRT PAC can appropriately select patients for device therapy and results in substantial savings for the NHS. It avoids unnecessary interventions, thereby reducing pacing clinic appointments, battery changes and the potential need for device revisions. Adopting this clinic in more centres across the NHS has the potential to save over £39 million, especially important in an already burdened healthcare system.
